# Cooperation and constraint: the complex association of caspase-8 and cFLIP

**DOI:** 10.1038/s41418-026-01785-8

**Published:** 2026-07-27

**Authors:** Betsaida Bibo-Verdugo, Guy S. Salvesen

**Affiliations:** 1 Centro de Investigaciones Biológicas del Noroeste S.C., La Paz, Mexico; 2 Sanford Burnham Prebys, La Jolla, CA USA

**Keywords:** Cell death and immune response, Proteolysis

**Fig. 1:**
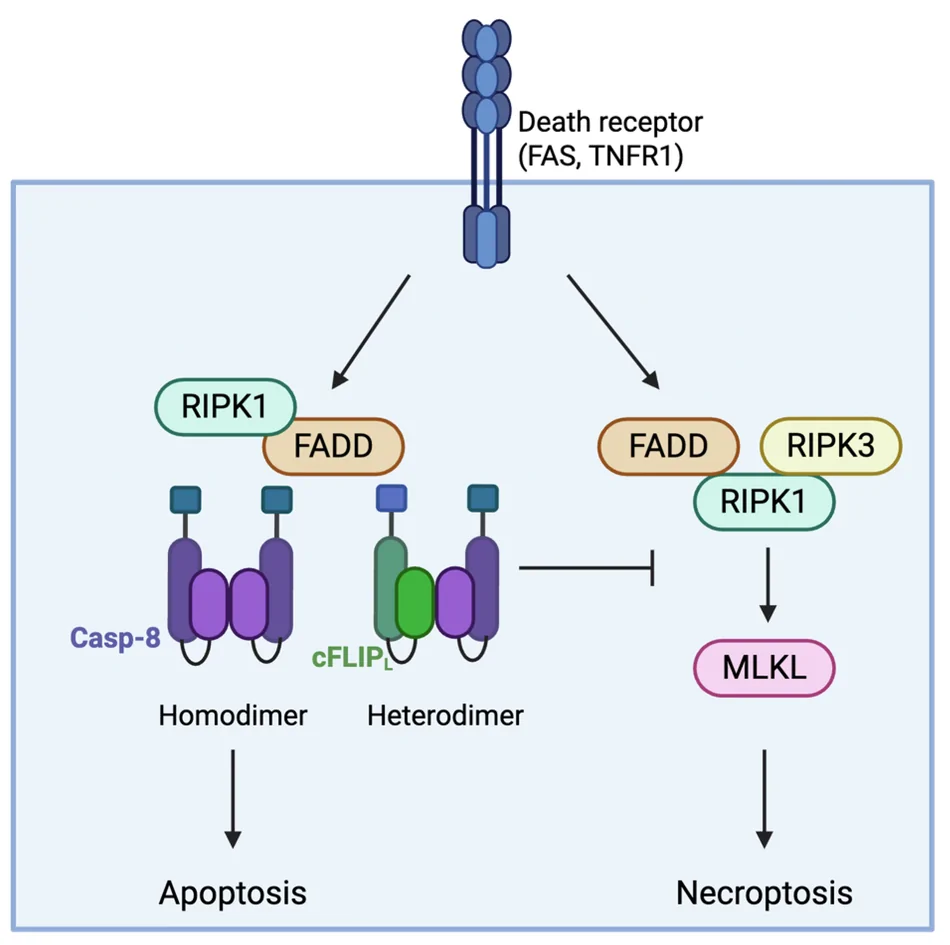
Cell death pathways influenced by the dimerization state of caspase-8.

Caspases exert their function by cleaving proteins, primarily self-cleavage as part of their maturation in cell death signaling complexes, and cleavage of other caspases to engage downstream cell death signaling pathways. Caspase-8 has a unique place in cell death decisions because it can both promote apoptosis and prevent necroptosis (Fig. 1), thereby engaging opposing pro-and anti-inflammatory pathways [1]. Mechanistically-speaking, these opposing outcomes depend on interaction with the adaptor protein FADD (Fas-Associated Death Domain protein), dimerization state and self-cleavage. Downstream from recognition of death ligands by their cognate receptors, including Fas and TNFR1 (Tumor Necrosis Factor Receptor 1), latent caspase-8 is activated by dimerization via recruitment to the oligomeric platform known as the death inducing signaling complex (DISC). Once activated, a caspase-8 homodimer can cleave itself at position Asp387 in mouse, or Asp374 or Asp384 in human. This processing event splits the catalytic domain into two subunits, resulting in a more active and stable conformation that is crucial for its proapoptotic function in the context of homodimerization [2]. Adjacent to the *Casp8* genetic locus is the gene encoding the pseudocaspase cFLIP (Cellular FLICE-like Inhibitory Protein), *Cflar*. A variant of this protein (cFLIP_L_) contains the same protein fold as caspase-8 but lacks the catalytic residues. Increased concentration of cFLIP_L_ favor the formation of caspase-8/cFLIP_L_ heterodimers, which inhibit caspase-8 proapoptotic function but can promote its anti-necroptotic function [3]. When caspase-8 partners with cFLIP_L_, intersubunit linker proteolysis is not required. In vitro, the heterodimer is an attenuated enzyme [4]. Thus, cFLIP_L_ can activate caspase-8 by dimerization to generate a heterodimer with different substrate specificity than the homodimeric caspase-8, which has an apoptotic role.

Elucidating the consequences of caspase-8 influence on death decisions heavily relies on genetic mouse models. Mice lacking caspase-8 or expressing inactive caspase-8 do not survive embryonic development. Although apoptosis is compromised, necroptosis is unrestrained [5–7]. Mutations that prevent necroptosis (deletion of *Mlkl* or *Ripk3*) have a rescuing effect on the lethal phenotype [8, 9]. On the other hand, mutations that prevent full caspase-8 activation cause apoptosis insufficiency and produce lymphoproliferative disease (LPR) in mice and autoimmune lymphoproliferative syndrome (ALPS) in humans [10]. Although self-processing of caspase-8 is considered important for apoptosis, self-processing-deficient caspase-8 (D384A) mice do not develop LPR.

A recent paper by Newton and colleagues addresses this somewhat confusing conundrum using mice with specific mutations in *Casp8* and *Cflar* bred together [11]. In a *Casp8* WT background, *Cflar* loss-induced lethality is rescued by deletion of *Mlkl*. However, while embryos resulting from a cross of mice bearing an inactivation of the caspase-8 self-cleavage site (D384A) with *Mlkl*^-/-^ mice are viable, concomitant loss of *Cflar* leads to perinatal lethality, atrophy of small intestine and thymus, and upregulation of cytokine and chemokine transcripts. The culmination of this work indicates that cleavage-deficient caspase-8 D387A can form active heterodimers with cFLIP_L_ to promote apoptosis, whereas caspase-8 D387A homodimers cannot sustain this function. This model is supported by a previous work where ablation of caspase-8 homodimers was achieved by mutations in the pro-domain [12]. Importantly, this outcome depends on restraining the necroptotic pathway, highlighting the fact that dominance of cell death paradigms depends on the dynamic competition of cell death signaling proteins. Caspase-8 D387A homodimers could not cleave caspase-3 to trigger extrinsic apoptosis, supporting prior biochemical findings that the first cleavage in the DISC must be a self-cleavage to generate a conformation of caspase-8 competent for downstream signaling. Over the years, the supposed function of cFLIP_L_ has changed from apoptosis inhibitory protein to modulator of caspase-8 specificity, culminating in the latest publication that cleverly uses mouse genetics to reveal the mechanism that this pseudocaspase utilizes to modulate cell death signaling pathways.

Notwithstanding the attractive answers to how caspase-8 activity is controlled in mice to signal distinct cell death outcomes we note that humans contain a caspase-8 paralog in the genomic region between *Casp8* and *Cflar*. Caspase-10 is absent in mice, and although it can also be activated by homodimerization or heterodimerization the resulting enzymes can promote apoptosis but cannot block necroptosis in vitro [13]. While the core apoptotic machinery is functionally conserved across mammals [14], central components of the necroptotic pathway have been deleted or show inactivating mutations in several mammalian lineages [15]. From an evolutionary perspective, it appears that the apoptotic pathway is indispensable and that genes regulating necroptosis are under extreme selection, resulting in their loss in some mammal orders and we look forward to future investigations of this interesting phenomenon.
